# A Mixed-Method Study of Unhealthy Feeding Practices Among Mothers of Children Aged 6–23 Months from an Internally Displaced Person Camp, Kayin State, Myanmar

**DOI:** 10.3390/nu18162680

**Published:** 2026-08-17

**Authors:** Htet Zaw Shein, Napaphan Viriyautsahakul, Shuhei Nomura, Wandee Sirichokchatchawan

**Affiliations:** 1 College of Public Health Sciences, Chulalongkorn University, Bangkok 10300, Thailand; 2 Global Health Policy Lab, International Research Institute of Disaster Science (IRIDeS), Tohoku University, 468-1 Aoba, Aramaki, Aoba-ku, Sendai 980-8572, Miyagi, Japan; 3 Global Health Policy Lab, Graduate School of Medicine, Tohoku University, 2-1 Seiryo-machi, Aoba-ku, Sendai 980-8575, Miyagi, Japan; 4 Department of Health Policy and Management, School of Medicine, Keio University, 35 Shinanomachi, Shinjuku-ku, Tokyo 160-8582, Japan; 5 Center of Excellence in Diagnosis and Monitoring of Animal Pathogens (DMAP), Bangkok 10330, Thailand

**Keywords:** complementary feeding, unhealthy feeding practices, nutrition transition, internally displaced persons, infant and young child feeding, maternal decision-making autonomy, Myanmar

## Abstract

Background/Objectives: Conflict-driven displacement can disrupt food environments, health services, and caregiver practices, increasing the risk of unhealthy feeding behaviors among young children. Evidence from ethnic internally displaced populations (IDPs) in Myanmar remains scarce. This study examined the prevalence and determinants of unhealthy feeding practices among IDP mothers of children aged 6–23 months. Methods: A sequential explanatory mixed-method study was conducted among 617 mothers in Karen ethnic IDP camps in Myanmar. Structured interviews were used to assess mother-reported zero vegetable or fruit consumption (ZVF) and unhealthy food consumption (UFC) among children, and multivariable logistic regression identified associated factors. Subsequently, six focus group discussions explored contextual influences on feeding practices and were analyzed via thematic analysis. The findings were integrated using a joint display approach. Results: A total of 29.2% of children experienced ZVF, while 57.5% consumed unhealthy foods. Mothers who attended fewer than the recommended antenatal care (ANC) visits were more likely to report both ZVF (AOR = 1.968, 95% CI: 1.297–2.988) and UFC (AOR = 1.830, 95% CI: 1.265–2.649). Higher maternal decision-making autonomy was also associated with increased odds of ZVF (AOR = 1.757, 95% CI: 1.031–2.995) and UFC (AOR = 1.606, 95% CI: 1.040–2.481). Other associated factors included child age, household income, family structure, paternal employment, and household dietary practices. Qualitative findings identified six themes influencing feeding practices: “familiarity breeds acceptance”, “traditional beliefs and food restrictions”, “constrained local food environments”, “time constraints and convenience feeding”, “preference for sweet foods”, and “disrupted access to health and nutrition services”. Conclusions: Unhealthy feeding practices were common in this displaced population. Greater maternal decision-making autonomy was not necessarily associated with healthier feeding practices, highlighting the importance of nutrition literacy and supportive food environments. Community-based nutrition interventions may help improve complementary feeding practices in conflict-affected settings.

## 1. Introduction

Globally, an estimated 148 million children under the age of five are stunted, while 37 million are affected by overweight, highlighting the double burden of malnutrition with the coexistence of undernutrition and overnutrition. This burden is not evenly distributed across populations, disproportionately affecting children living in fragile and conflict-affected settings [1,2]. These disparities reflect nutrition inequities: the systematic and avoidable differences in nutrition-related outcomes driven by unequal access to resources and opportunities. Such inequities are not merely the result of individual practices but are deeply rooted in broader social, economic, and political conditions, affecting marginalized populations and contributing to persistent racial, ethnic, and geographic health disparities [3,4,5].

In Myanmar, longstanding political processes have shaped the country’s health disparities. The concept of structural violence offers a lens for understanding these inequities, highlighting how governance, legal frameworks, and economic policies constrain communities from achieving their full health potential [6]. The process of Burmanization presents a key manifestation of these disparities. The 1982 Citizenship Law has further entrenched these disparities by stratifying citizenship and limiting access to rights and essential services for non-Bamar groups [7,8]. Consequently, ethnic minority populations, including the Kayin, have experienced chronic underinvestment in healthcare, education, and nutrition services, leading to the intergenerational vulnerabilities reflected in present-day health outcomes [2,9].

More recently, the 2021 Military Coup has further exacerbated these inequities by disrupting public systems and services, intensifying pre-existing structural disparities rather than constituting an isolated crisis [10,11]. The collapse of Myanmar’s healthcare system was driven not only by administrative disruption, but also by targeted attacks on healthcare workers participating in the Civil Disobedience Movement. This has had profound implications for maternal and child health services, including immunizations and childhood nutrition programs [10,12]. Only 4.9% of the children in this study had completed recommended immunization schedules, reflecting a broader governance vacuum and the severe disruption of essential public health services. Consequently, these conditions have widened existing service gaps and reinforced patterns of exclusion rooted in longstanding structural inequalities among minority populations.

The Kayin ethnicity, one of the largest ethnic minority groups in Myanmar, represents approximately 7% of the national population. This heterogeneous group comprises multiple subgroups, including Sgaw and Pow, each with distinct linguistic, cultural, and religious characteristics [7,13]. Kayin State has been the site of one of the longest-running civil conflicts, characterized by decades of tension between the Myanmar Military Services and Ethnic Armed Organizations [7,14]. The 2021 political crisis reignited active hostilities in the region, indicating that communities are not transitioning toward recovery, but instead experiencing compounded stressors, including active conflicts, cyclical displacement, and economic instability [12,15]. In this context, internally displaced person (IDP) camps present highly constrained environments where access to resources, essential services, and social support systems is severely limited [16,17].

This study is informed by a risk environment framework, which conceptualizes health behaviors, including child feeding, as shaped by interacting environmental domains [18,19]. Particularly in IDP camp settings, marginalized ethnic groups face multiple, overlapping risks. Geographic isolation and limited access to markets, often requiring long travel times, are common physical risks [15,17]. Economic risks include constrained household incomes and reliance on humanitarian assistance, while social risks emerge with the persistence of cultural norms, including food taboos [20,21,22]. Lastly, political risks arise from weakened governance and disrupted healthcare services, limiting access to health education and preventive care [10,11]. These intersecting risks create an unfavorable environment for caregivers to provide optimal nutrition practices, compromising dietary quality among children in displaced and conflict-affected settings.

The Nutrition Health Disparities Framework (NHDF) guides the further contextualization of how these environmental constraints translate into individual behaviors. As part of broader interpersonal and behavioral domains, maternal decision-making and paternal support do not operate in isolation but are shaped by structural and environmental conditions that influence available choices [23]. Therefore, unhealthy food consumption can also be a response to environments where nutrient-poor foods are often more accessible, affordable, and socially normalized than healthier alternatives, rather than being due to individual preference, in resource-constrained settings [19,24]. This framework examines the synergistic relationship between biological, behavioral, physical, sociocultural, and healthcare system domains [4,25].

Although the global literature highlights nutrition disparities in conflict-affected settings, empirical evidence from Myanmar, particularly among ethnic minority IDPs, remains limited, and important ethnic, geographic, and contextual variations are often overlooked. Integrated approaches are therefore needed to examine how structural and environmental factors interact to shape feeding practices among ethnic minority populations. This gap is particularly important in the current displacement context, where caregiving practices, access to food, and healthcare services are often disrupted and no longer follow usual patterns. Therefore, this study employs a sequential explanatory mixed-method design to examine the prevalence and determinants of zero vegetable or fruit consumption (ZVF) and unhealthy food consumption (UFC) among mothers of children aged 6–23 months in an IDP camp in Kayin State, Myanmar.

## 2. Materials and Methods

**Figure 1:**
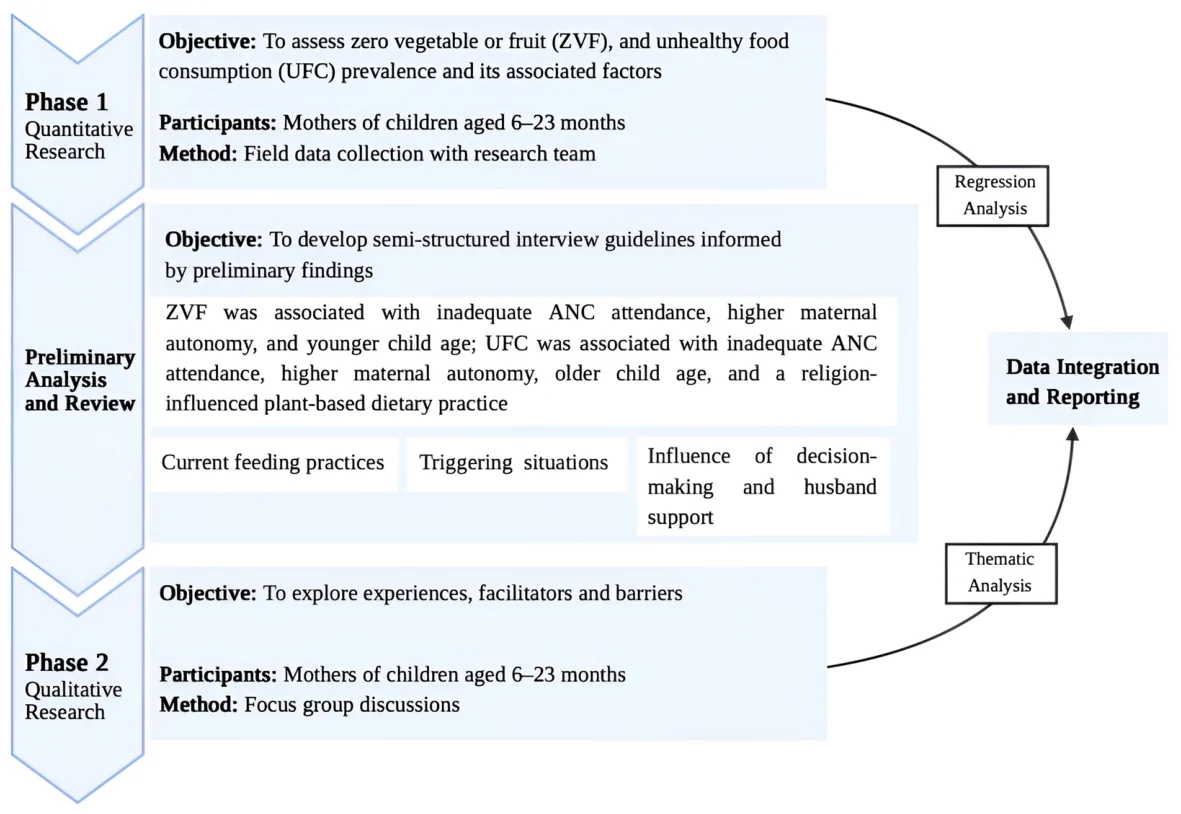
Schematic diagram of the sequential explanatory mixed-method design. This figure illustrates the sequence of a study with a sequential explanatory design, involving each objective and then expanded into qualitative explanation based on the quantitative phase of the study. The authors created this diagram using BioRender (Toronto, ON, Canada; https://www.biorender.com/ accessed on 1 August 2026).

This study used a sequential explanatory mixed-method design [26]. A cross-sectional survey was conducted in the quantitative phase, and the findings informed the development of a semi-structured interview guide for focus group discussions in the qualitative phase (Figure 1). The overall study spanned from May 2023 to April 2024, comprising a quantitative data collection phase (May–September 2023) and a qualitative data collection phase (December 2023–April 2024). The Aye Linn Myat Shin IDP camps in the Myaing Gyi Ngu Ethnic Special Zone, Kayin State, were purposively selected as the study site. These camps host approximately 2485 Kayin IDPs displaced by the 2021 humanitarian crisis. They are characterized by political instability, dependence on humanitarian aid, and strong traditional and religious influences on child-feeding practices.

### 2.1. Sample

The IDP registry and the rural health center population profile identified 675 mothers with children aged 6–23 months residing in the study area during the quantitative data-collection period. The two sources were cross-checked with camp committees before data collection to verify the completeness and accuracy of the population list. After applying the study eligibility criteria, 625 mothers were eligible for participation. The eligibility criteria were (1) being the primary caregiver of a child aged 6–23 months; (2) being able to communicate in Sgaw or Pwo Karen; (3) providing informed consent; and (4) having lived with their husband for at least two consecutive years, because the study included measures of maternal intrahousehold decision-making autonomy and husband supportive behavior.

**Figure 2:**
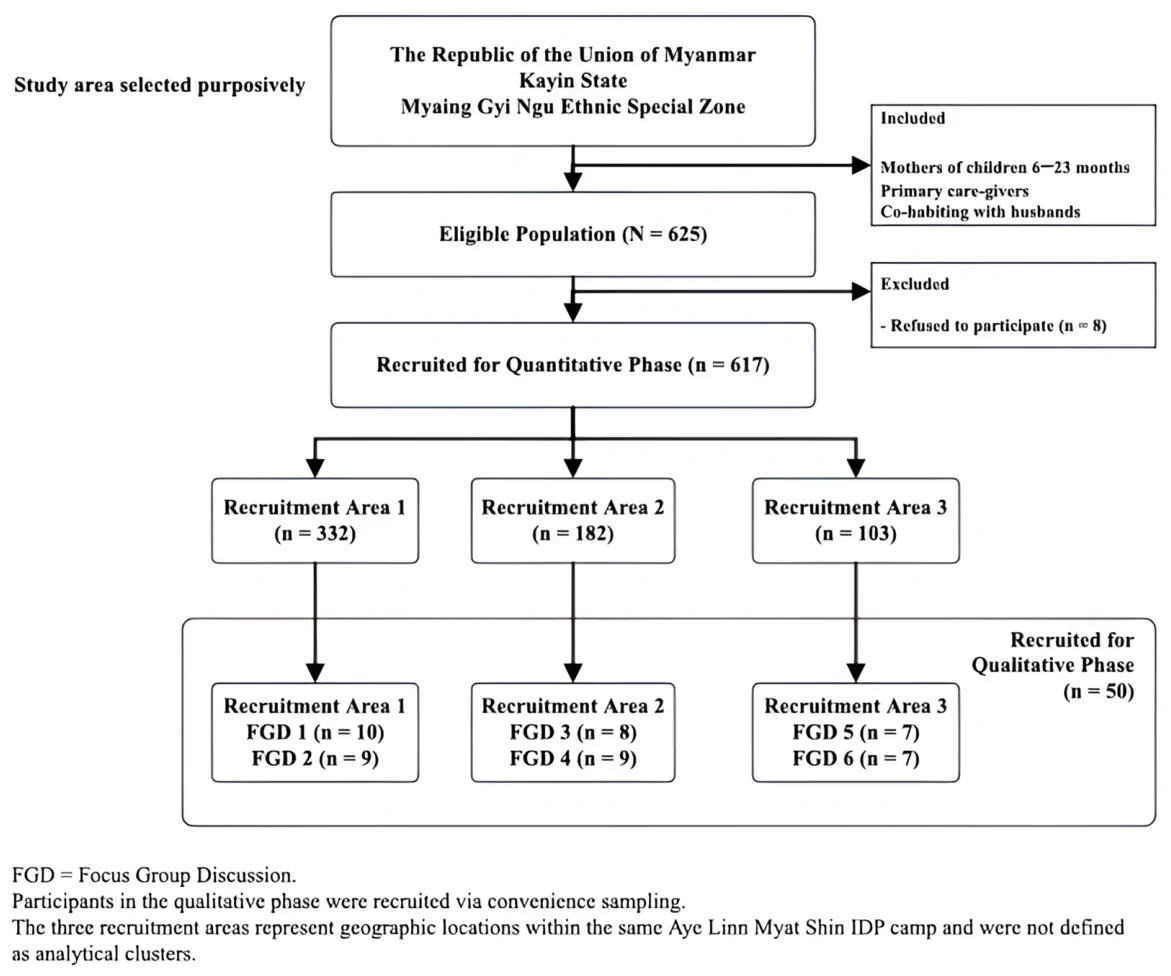
Sampling flow chart for both phases. The authors created this diagram using Microsoft Visio for Microsoft 365 (Microsoft Corporation, Redmond, WA, USA).

All 625 eligible mothers were invited to participate, representing a census of the eligible study population rather than a sampled subset. Quantitative data collection was conducted over multiple growth monitoring and promotion (GMP) sessions. Mothers who were unavailable during the initial data-collection session were approached again during subsequent sessions whenever possible. A total of 8 eligible mothers declined to participate, resulting in a final quantitative sample of 617 mothers (Figure 2).

Following preliminary quantitative analysis, 50 mothers were convenience-sampled from among the quantitative survey participants who voluntarily agreed to participate in the qualitative phase. The participants were drawn from the three geographical recruitment areas within the IDP camp. The qualitative interview guide was subsequently refined based on the preliminary quantitative findings. Camp committees, local health staff, and border guard forces provided logistical support, including facilitating access to the study locations and communicating the data-collection schedule. They were not involved in determining participants’ eligibility, obtaining informed consent, conducting interviews, or accessing individual responses. Participants were informed that participation was voluntary and that declining or withdrawing from the study would not affect their access to healthcare, humanitarian assistance, or other services.

### 2.2. Phase I: Quantitative Study

Data collection was conducted by a field research team composed of 11 local nutrition promotion volunteers. The volunteers received training on the study protocol, informed consent, confidentiality, standardized interviewing procedures, and key study indicators, and were supervised by a public health assistant. Data were collected using KOBO Toolbox/Collect, an open-source online data collection tool [27]. All eligible mothers were invited to each camp’s distribution site, with the support of camp committees and local health staff. Quantitative interviews were conducted in a designated separate area at the distribution site to prevent responses from being overheard. Only the participant and a trained data collector were present during the interview, and individual responses were not shared with camp committees, health personnel, or security personnel. Each interview lasted approximately 30 min. Each participant received a low-value, non-cash hygiene item, a bar of antibacterial soap valued at approximately 1500 MMK (USD 0.30), as a token of appreciation for the time required to complete the interview. The item was selected because of its practical value and minimal monetary worth, thereby minimizing the potential for undue influence. Participants were informed that their participation was voluntary and that declining or withdrawing would not affect their access to healthcare, humanitarian assistance, or other services. The compensation procedure was included in the protocol approved by the ethics review committee. The field supervisor conducted daily briefings with the team and checked the web-based database for the completeness and accuracy of data.

### 2.3. Measures

For the quantitative phase, data were collected using a modified structured questionnaire comprising five main parts: (1) general characteristics; (2) household characteristics; (3) maternal intrahousehold decision-making autonomy; (4) husband supportive behavior; and (5) feeding practices, specifically ZVF and UFC.

### 2.4. General and Household Characteristics

Mothers’ general characteristics were self-reported and included age, literacy status, employment, history of antenatal care (ANC), and counseling on infant and young child feeding (IYCF). The number of ANC contacts was categorized according to the WHO’s recommended minimum frequency. In addition, husbands’ literacy and employment status were collected. Child characteristics included age, sex, immunization status, and history of malnutrition. Household characteristics included household type (nuclear or extended); household head (father, mother, or other); monthly income (above 250,000 MMK or ≤250,000 MMK); and household dietary practice. Household dietary practice was classified according to each household’s reported dietary practice as either (1) conventional dietary practice or (2) religion-influenced plant-based dietary practice. Classification was made at the household level rather than according to geographical recruitment area, and both dietary practices were represented across the three recruitment areas within the IDP camp.

### 2.5. Maternal Intrahousehold Decision-Making Autonomy

Maternal intrahousehold decision-making autonomy was assessed using the Composite Index of Women’s Autonomy (CIWA) [28], comprising seven items across four domains: mobility, social participation, health-seeking, and financial decision-making. Each item was scored as 2 for decisions made independently, 1 for decisions made jointly with the husband, and 0 for no participation in the decision. Item scores were summed to obtain a total score ranging from 0 to 14, with higher scores indicating greater decision-making autonomy. The scale demonstrated good internal consistency in the current sample (Cronbach’s α = 0.821), and no missing data were observed. As no validated cutoff was available, the sample mean was used as an operational cutoff, with scores below the mean classified as lower autonomy and scores at or above the mean as higher autonomy. Sensitivity analysis was conducted using the sample median as an alternative cutoff, and the original continuous autonomy scores are presented in Table A1.

### 2.6. Husband Supportive Behavior

Husband supportive behavior in child feeding was assessed using a six-item three-point Likert scale adapted from a study conducted in South Ethiopia [29]. The scale covered four domains: (1) decision-making regarding food items; (2) participation in daily household chores; (3) problem-solving related to feeding difficulties; and (4) participation in nutrition promotion activities. Each item was scored as 0 for never, 1 for sometimes, and 2 for every time involvement, yielding a total score ranging from 0 to 12, with higher scores indicating greater husband support. In the present sample, the scale demonstrated acceptable internal consistency (Cronbach’s α = 0.752), and no missing data were observed. As no validated cutoff is available, the sample mean was used as an operational cutoff, with scores below the mean classified as lower husband support and scores at or above the mean as higher husband support. Sensitivity analysis using the sample median as an alternative cutoff, and the original continuous husband-support score are presented in Table A1.

### 2.7. Sensitivity Analysis

Sensitivity analyses using alternative operationalizations of maternal intrahousehold decision-making autonomy and husband supportive behavior generally supported the primary findings. Associations between maternal autonomy and both ZVF and UFC were consistent across mean-based, median-based, and continuous-score specifications. Husband supportive behavior remained unassociated with both ZVF and UFC across these specifications (Table A1). For ZVF, a statistically selected model showed improved calibration compared with the expanded theory-informed model (Hosmer–Lemeshow *p* = 0.103 versus 0.005), while the direction and overall interpretation of the principal associations were generally consistent across the two model specifications (Table A2).

### 2.8. Unhealthy Feeding Practices

Both outcomes were defined according to the 2018 WHO and UNICEF IYCF indicators (Appendix A). ZVF was defined as no consumption of vegetables or fruits during the previous day. Assessment of ZVF included vitamin A-rich yellow or orange vegetables and fruits, dark green leafy vegetables, and other vegetables and fruits, based on a locally adapted food list. UFC was defined as the consumption of selected sentinel unhealthy foods during the previous day, regardless of quantity, including candies or candied fruit, frozen treats, sweetened cakes, fried confections, and instant noodles [30]. To facilitate recall, a locally adapted visual food list of commonly consumed items was used during the interviews.

### 2.9. Statistical Analysis

Data were analyzed using the IBM SPSS Statistics, version 29.0 (IBM Corp., Armonk, NY, USA). Descriptive statistics were presented as means, medians, standard deviations, and interquartile ranges for continuous variables, and as frequencies and percentages for categorical variables. Associations with ZVF and UFC were initially examined using univariable logistic regression. Candidate variables for the multivariable models were selected based on the study’s conceptual frameworks, prior evidence, study objectives, and plausible confounding relationships; additional variables with *p* < 0.20 in the univariable analyses were also considered for inclusion. Variables identified a priori as potential confounders were retained irrespective of their statistical significance in the univariable analysis. Separate multivariable logistic regression models were fitted for ZVF and UFC. Model calibration was assessed using the Hosmer–Lemeshow goodness-of-fit test, explanatory power was assessed using Nagelkerke R^2^, and multicollinearity was assessed using variance inflation factors. Because the expanded theory-informed ZVF model showed evidence of suboptimal calibration, a statistically selected model based on the original core covariate specification was additionally examined as a sensitivity analysis to assess the robustness of the effect estimates and model calibration (Table A2). As all variables included in the final models were categorical, assessment of linearity in the logit was not applicable. Statistical significance was set at *p* < 0.05, and results were reported as adjusted odds ratios (AORs) with 95% confidence intervals.

### 2.10. Phase II: Qualitative Study

Six focus group discussions (FGDs) were conducted with 50 mothers selected by convenience sampling from among participants in the quantitative survey who subsequently volunteered to participate in the qualitative phase (8–10 participants per group). Thus, the qualitative participants were drawn from the quantitative survey sample rather than recruited independently. The semi-structured interview guide, provided as Appendix C, was developed based on the preliminary quantitative findings to further explore and contextualize factors associated with child-feeding practices, particularly ZVF and UFC. The guide covered three main areas: (1) current child-feeding practices; (2) contextual factors related to ZVF and UFC; and (3) maternal decision-making and husband support in relation to child feeding. Participants were invited face-to-face, and each FGD lasted approximately 30–45 min. Each FGD was conducted in a private area at the camp community gathering site. Only study participants and members of the research team were present; community leaders, health workers, security personnel, and non-participants were not present during the discussions. Participants were identified by unique study identification numbers in field notes and transcripts, and audio recordings and study data were accessible only to the research team. Data collection continued until thematic saturation was achieved. Saturation was assessed by the research team through an ongoing review of emerging codes after each FGD, and was considered to have been reached when no new codes or themes emerged in subsequent discussions. At the end of each FGD, the facilitator summarized the main points discussed, and participants were invited to correct, clarify, or add information. Participants confirmed that the summary accurately reflected their views. All discussions were audio-recorded with prior consent, and unique identification numbers were used in field notes to protect confidentiality.

### 2.11. Reflexivity

The research team had previously engaged with the Kayin community through nutrition programs, which facilitated rapport and participant recruitment. However, the presence of experienced nutrition specialists may have introduced social desirability bias, as displaced mothers may have felt pressure to report favorable feeding practices in line with perceived expectations. Mitigation measures included emphasizing confidentiality, encouraging honest responses, and using neutral, non-judgmental questioning techniques.

### 2.12. Data Analysis

The translation team, led by the principal researcher, transcribed the discussions verbatim in the local ethnic language, translated them into English, and triangulated the content with field notes. A deductive thematic analysis was conducted using NVivo (Release 1.7, QSR International, Doncaster, VIC, Australia, 2023), with the preliminary quantitative findings guiding the initial coding framework while allowing for additional themes to emerge from the data. The first author performed the initial coding, organized the data into meaningful categories, and developed themes, which were then reviewed and validated through discussion with the second author to address any discrepancies or ambiguities (Table A3). Data triangulation was achieved by integrating findings from focus group discussions with quantitative survey results, allowing for cross-validation and deeper interpretation of feeding practices within the displacement context.

## 3. Results

### 3.1. Quantitative Findings

#### 3.1.1. General Characteristics of Mothers, Children Aged 6–23 Months, and Household Characteristics

While reading and writing ability was relatively high (69.0%), two-thirds of mothers were unemployed (66.3%). Approximately 37.4% attended at least eight ANC visits, and 58.5% received postnatal IYCF counseling. Most mothers reported high decision-making autonomy (84.1%); however, only 52.7% received fair husband support in child feeding. Among children, the majority were aged 12–23 months (84.3%), and alarmingly, only 4.9% had completed the recommended immunization schedule. Furthermore, 60.1% of households followed religion-influenced plant-based dietary practice patterns (Table 1).

#### 3.1.2. Prevalence and Determinants of ZVF

Overall, 29.2% of children experienced ZVF. Among maternal factors, ZVF was more common among children whose mothers did not meet the WHO-recommended number of ANC visits (33.7%) (Table A4). In the adjusted analysis, these children had nearly twice the odds of ZVF compared with those whose mothers met the recommended level (AOR = 1.968; 95% CI: 1.297–2.988). Higher maternal decision-making autonomy was also associated with ZVF; 30.8% of children whose mothers reported higher autonomy experienced ZVF, and the association remained significant after adjustment (AOR = 1.757; 95% CI: 1.031–2.995).

Child age was also strongly associated with ZVF. The prevalence was higher among children aged 6–8 months and 9–11 months than among those aged 12–23 months. Compared with the oldest age group, children aged 6–8 months had substantially higher odds of ZVF (AOR = 7.570; 95% CI: 3.594–15.945), while those aged 9–11 months also had higher odds (AOR = 2.806; 95% CI: 1.530–5.146).

At the household level, ZVF was associated with extended household structure, lower household income, and religion-influenced plant-based dietary practice. In the adjusted model, the odds of ZVF were higher among children living in extended households (AOR = 3.280; 95% CI: 1.006–6.698), lower-income households (AOR = 2.840; 95% CI: 1.291–6.248), and households following a religion-influenced plant-based dietary practice (AOR = 1.840; 95% CI: 1.249–2.710) (Table 2).

Model calibration for the expanded theory-informed ZVF model was suboptimal (Hosmer–Lemeshow *p* = 0.005). However, a statistically selected model showed improved calibration (*p* = 0.103), while the direction and overall interpretation of the principal associations were generally consistent (Table A2).

#### 3.1.3. Prevalence and Determinants of UFC

UFC was observed among 57.5% of children. Among maternal factors, a higher prevalence of UFC was observed among children whose mothers did not meet the WHO-recommended number of ANC visits. In the adjusted analysis, these children had higher odds of UFC than those whose mothers met the recommended level (AOR = 1.830; 95% CI: 1.265–2.649). Similarly, the prevalence of UFC was 59.5% among children whose mothers reported higher decision-making autonomy (Table A4), which remained independently associated with UFC after adjustment (AOR = 1.606; 95% CI: 1.040–2.481).

Child age was also associated with UFC. The highest prevalence was observed among children aged 12–23 months (61.2%). Compared with children aged 6–8 months, those aged 12–23 months had approximately three times the odds of UFC (AOR = 3.045; 95% CI: 1.468–6.318). A higher prevalence of UFC was also observed among children whose fathers were employed (60.8%) and among those living in households following a religion-influenced plant-based dietary practice (66.6%). Both factors remained associated with UFC in the adjusted model: paternal employment (AOR = 1.788; 95% CI: 1.133–2.822) and religion-influenced plant-based dietary practice (AOR = 2.566; 95% CI: 1.799–3.662) (Table 2).

### 3.2. Qualitative Findings

Six themes were identified across the six FGDs among 50 participants, as presented in Table A5. Themes 1 and 2 primarily provided context for ZVF, Themes 3–5 primarily provided context for UFC, and Theme 6 provided context for both outcomes. The distribution of each theme across the six FGDs is reported below, together with variations in participants’ experiences where identified. The development of initial codes, minor themes, and major themes is presented in Table A3.

#### 3.2.1. Theme 1: Familiarity Breeds Acceptance

Mothers described difficulties in introducing unfamiliar tastes and textures during the early complementary feeding period, particularly vegetables and semi-solid foods. This theme was identified in all six FGDs. Mothers commonly reported food refusal when complementary foods were first introduced, whereas acceptance tended to improve with repeated exposure and increasing familiarity. These accounts complement the quantitative finding that younger children had higher odds of ZVF by providing insight into feeding difficulties during the early complementary-feeding period (Table 3).

“It’s easier to feed 4–5 times for a child of 12–23 months because they become familiar with the taste and consistency of the complementary foods. At first, at the age of 6 months, he cried when I introduced soft or semi-solid food to him.”

(Participant 3, FGD 5).

“My kid usually denies it and spits out the steamed rice mixed with chopped and boiled carrot or cabbage because the taste is not too good.”

(Participant 5, FGD 4).

#### 3.2.2. Theme 2: Traditional Beliefs and Food Restrictions

Traditional beliefs and advice from family members shaped perceptions of foods considered appropriate for young children. This theme was identified in four of the six FGDs. Mothers described restrictions on particular vegetables and other foods, sometimes creating tension between nutrition advice and expectations from older household or community members. These findings complement the quantitative association between extended household structure and ZVF by providing context on how household members and food-related beliefs may influence feeding decisions (Table 3). However, the qualitative findings do not establish that these practices account for the statistical association with an extended household structure. Some mothers described being able to follow nutrition advice despite traditional expectations, indicating variation in the influence of household norms.

“My mother also makes me avoid feeding green vegetables to my baby because the green and leafy vegetables lead to constipation in children, as they believe in food taboos.”

(Participant 5, FGD 2).

“Since we have to depend on the camp committee for our living and shelter, I have to follow the food culture here.”

(Participant 2, FGD 4).

“But, when she (the grandmother) became aware of the importance of various food groups, she let me introduce the green leafy vegetables to her granddaughter”

(Participant 6, FGD 4).

#### 3.2.3. Theme 3: Time Constraints and Convenience Feeding

Time constraints related to household responsibilities, informal work, and community activities were described in all six FGDs. Mothers reported that limited time for preparing recommended complementary foods sometimes resulted in reliance on readily available foods for their children. These accounts expand the quantitative finding regarding paternal employment by highlighting broader household time and caregiving constraints, but they do not directly explain the association between paternal employment and UFC (Table 3).

“I usually do like that when I am so swamped with daily chores. I have no extra time to prepare those specific complementary foods for my kid. So, he must eat like us or ready-made food for his lunch. When we have a little extra money from my husband’s work, I sometimes buy ready-made snacks because they are convenient, especially when I am busy caring for the family.”

(Participant 3, FGD 2).

“When I am so swamped with daily chores, I have to buy cake or biscuits for my child. It is easier than preparing complementary foods.”

(Participant 1, FGD 4).

#### 3.2.4. Theme 4: Constrained Local Food Environments

Constraints in the local food environment were reported in five of the six FGDs. Mothers described geographical and financial barriers to obtaining diverse and nutritious foods, including long travel distances to markets and limited household resources. In contrast, nearby shops offered readily available shelf-stable foods. Some mothers also described exchanging humanitarian assistance items for food. These findings complement the quantitative association between lower household income and ZVF and expand the understanding of the broader food environment in which household dietary practices occur (Table 3). However, the qualitative data did not directly establish why religion-influenced plant-based dietary practice was associated with UFC.

“I bought some cake or biscuits and fed my child, especially after lunch. I can buy or exchange with some aid items at the nearby grocery shops”

(Participant 1, FGD 2).

“I had a lot of days that my child ate no vegetables or fruits because the bazaar is too far from here. So, I had to feed my child steamed rice mixed with oils and salts, instant noodles, or instant vermicelli.”

(Participant 5, FGD 1).

#### 3.2.5. Theme 5: Preference for Sweet Foods

Mothers described children’s increasing preference for sweet foods in all six FGDs. As children grew older, some increasingly requested sweet snacks and drinks and rejected other foods, making it more difficult for caregivers to maintain recommended feeding practices. Mothers also described crying or agitation when preferred foods were withheld. These accounts complement the quantitative finding that older children had higher odds of UFC by providing context on the development of food preferences with increasing age (Table 3).

“My child prefers sweet snacks. If I do not buy them, he cries and becomes upset. As he gets older, he knows exactly what he wants to eat.”

(Participant 3, FGD 4).

“I could feed complementary food less than two times because my baby preferred cake and snacks. Sweet cake and breastmilk are the main foods he usually consumes. He doesn’t like the complementary food I prepare.”

(Participant 2, FGD 1).

“Children love those attractive and sweet cakes or desserts. They are easy to prepare.”

(Participant 4, FGD 5).

The last theme focused on the limited access to child-feeding knowledge, which may lead mothers to adopt unhealthy alternatives.

#### 3.2.6. Theme 6: Disrupted Access to Health and Nutrition Services

Disrupted access to health and nutrition services was reported in three of the six FGDs. Mothers described reduced access to vaccination services, health personnel, and opportunities to receive child-feeding information following political and health-service disruptions. These accounts complement the quantitative finding that mothers who did not meet the recommended number of ANC visits had higher odds of both ZVF and UFC by illustrating broader disruptions in access to maternal–child health and nutrition services (Table 3). The qualitative findings provide contextual information on reduced opportunities for nutrition counseling rather than establishing ANC attendance as a direct mechanism influencing feeding practices.

“Since 2021, I’ve seen fewer staff at the hospital. He missed his up-to-date vaccination, and we no longer get proper advice on child feeding at the vaccination site.”

(Participant 1, FGD 3).

“During these days, my child has not even received vaccination. So, it is difficult to meet with midwives or lady health visitors to receive health and nutrition education.”

(Participant 4, FGD 6).

### 3.3. Integrated Findings from Quantitative and Qualitative Analyses

**Figure 3:**
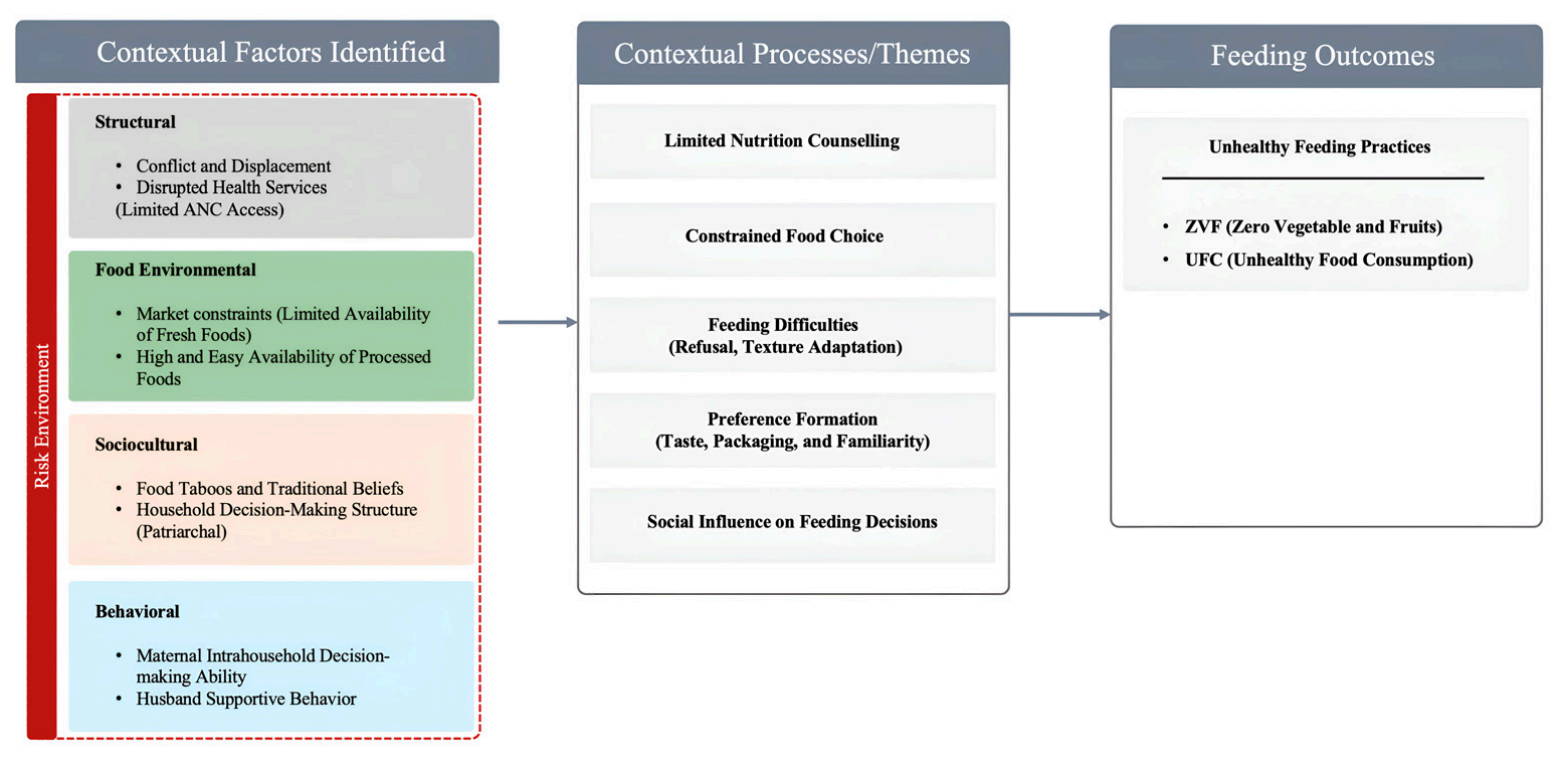
Conceptual organization of structural, food environmental, sociocultural, household, and behavioral factors associated with unhealthy feeding practices among IDP mothers. The authors created this diagram using BioRender (Toronto, ON, Canada; https://www.biorender.com/, accessed on 1 August 2026).

Figure 3 presents a conceptual interpretation of the integrated quantitative and qualitative findings, informed by the NHDF and the risk environment framework. The findings were organized across structural, food–environmental, sociocultural, household, and behavioral domains. Structural factors included conflict-related disruption and reduced access to health services; food–environmental factors included geographical and financial constraints on food access; sociocultural factors included traditional beliefs and food restrictions; and household and behavioral factors included maternal decision-making autonomy, husband support, caregiving demands, and child-feeding experiences. The contextual processes presented in the figure were derived primarily from the qualitative findings and are shown as interpretive relationships rather than empirically tested mediating or causal pathways.

The integrated findings are further summarized in the joint display (Table 3). Complementarity was observed for the associations involving child age, extended household structure, lower household income, and inadequate ANC visits, for which the qualitative findings provided contextual insight into the corresponding quantitative associations. Expansion was observed for paternal employment and household dietary practices, where the qualitative findings provided broader contextual information without directly explaining the quantitative associations. Silence was identified for maternal intrahousehold decision-making autonomy because this quantitative association was not represented by a corresponding qualitative theme. No substantial divergence between the quantitative and qualitative findings was identified.

## 4. Discussion

This study identified a substantial burden of unhealthy child-feeding practices among mothers of children aged 6–23 months living in an IDP setting, with 29.2% of children experiencing ZVF and 57.5% consuming selected sentinel unhealthy foods. The mixed-method findings indicate that these practices are embedded within a combination of child developmental factors, household circumstances, sociocultural influences, food environment constraints, and disrupted access to maternal and child health services. The qualitative findings complemented several quantitative associations by providing insight into mothers’ feeding experiences, while also expanding the understanding of broader contextual influences that were not fully captured by the quantitative measures.

The prevalence of ZVF in this study was 29.2%, lower than the reported Myanmar national estimate of 55.6% [31]. Differences in survey period, population characteristics, geographical setting, season, and measurement procedures should be considered when interpreting this comparison. More importantly, the qualitative findings showed that a relatively lower prevalence of ZVF did not necessarily indicate an unrestricted or nutritionally adequate food environment. Mothers described difficulties obtaining diverse and nutrient-rich foods, particularly when access depended on distant markets, household resources, and the availability of foods within the displacement setting. Similar constraints on dietary diversity have been reported in other conflict-affected populations [32,33]. Thus, ZVF prevalence should be interpreted within the broader context of constrained food access rather than as an indicator of overall dietary adequacy.

In contrast, more than half of the children consumed selected sentinel unhealthy foods. In displacement settings, high UFC may reflect constrained food environments in which inexpensive, convenient, and shelf-stable foods are readily available relative to more nutritious alternatives [34]. This pattern may be relevant to the broader nutrition transition reported in resource-constrained settings, although the present cross-sectional study did not assess changes in food environments or dietary patterns over time. Given the historically high burden of child undernutrition in Kayin State [35], frequent consumption of unhealthy foods may contribute to future nutrition-related risks associated with the double burden of malnutrition. However, current anthropometric indicators of stunting, wasting, overweight, or obesity were not assessed; therefore, this study does not demonstrate the coexistence of undernutrition and overnutrition [36].

Child age showed a particularly strong relationship with feeding practices. Children aged 6–8 months had substantially higher odds of ZVF, whereas UFC was more prominent among older children. The qualitative findings complemented these associations. Mothers described food refusal, unfamiliarity with new tastes and textures, and difficulties introducing complementary foods during the early feeding period, consistent with challenges reported during the transition to complementary feeding [37]. In contrast, mothers of older children described increasingly established food preferences and requests for sweet foods. Greater exposure to different foods as children get older may therefore alter the nature of feeding challenges, from difficulties introducing appropriate complementary foods in early infancy to managing preferences for less healthy foods later in childhood [38,39]. These age-related patterns support the importance of tailoring feeding guidance to different stages of complementary feeding.

Household structure was also associated with ZVF, with children living in extended households experiencing higher odds than those in nuclear households. The qualitative findings provided complementary insight into this association through accounts of traditional beliefs, food restrictions, and the influence of other household members on feeding decisions. Mothers described situations in which advice from older relatives shaped whether particular foods were considered appropriate for young children. Similar intergenerational influences on complementary feeding have been reported in other cultural settings [40,41]. These findings suggest that child-feeding decisions may extend beyond the mother alone, particularly in extended households where grandparents, husbands, elders, and other family members participate in decisions about food and childcare. Nutrition promotion approaches that engage influential household members alongside mothers may therefore be particularly relevant in such settings [42]. Household economic and food environment conditions provided another important layer of explanation. Lower household income was associated with ZVF, while qualitative accounts described financial barriers, long travel distances to markets, and limited local availability of nutritious foods. Theme 4 therefore complemented the quantitative income finding while also expanding the understanding of the broader food environment. Mothers described easy access to convenient and shelf-stable foods, but greater difficulty obtaining a diverse range of fresh and nutrient-rich foods. Such circumstances illustrate how food choice in displacement settings may be constrained not only by household resources, but also by what foods are practically available and accessible within the local environment [34].

UFC was additionally associated with paternal employment. The qualitative findings did not directly explain this association but expanded the understanding of the household circumstances in which convenience feeding occurred. Mothers described substantial time demands from childcare, household responsibilities, informal work, and community activities, sometimes resulting in reliance on readily available foods when preparation time was limited. These experiences are consistent with the concept of time constraints influencing household food preparation and convenience-food use [43,44]. Although paternal employment may be related to household routines and purchasing opportunities, household income itself was not independently associated with UFC. The mixed-method findings therefore suggest that the relationship may involve broader household organization and caregiving circumstances rather than a simple pathway from paternal employment to increased purchasing power [44].

Maternal intrahousehold decision-making autonomy showed a different pattern of integration. Higher autonomy was associated with greater odds of both ZVF and UFC, but no corresponding qualitative theme directly explained this relationship. Rather than indicating that greater autonomy inherently leads to poorer feeding practices, the finding may reflect the context in which maternal decision-making occurs. Greater reported autonomy may represent greater responsibility for making child-feeding decisions without corresponding control over food availability, affordability, or other household resources. In this displacement context, mothers may therefore have greater authority to make feeding decisions while still choosing from a restricted set of feasible options shaped by limited household resources, constrained market access, local food availability, caregiving demands, and disrupted health and nutrition services. Thus, greater decision-making authority does not necessarily imply greater ability to translate preferred feeding decisions into practice when available choices are constrained [45]. Because no qualitative theme directly linked maternal autonomy with ZVF or UFC, these contextual factors should not be interpreted as a demonstrated mechanism underlying the quantitative association. The joint display therefore defines this finding as silence.

Household dietary practice was also associated with UFC. Children in households reporting a religion-influenced plant-based dietary practice had higher odds of UFC than those in households reporting conventional dietary practices. The qualitative findings expanded this result by illustrating the constrained food environment in which household dietary practices occurred, including limited market access and reliance on locally available shelf-stable foods. The findings should therefore not be interpreted as suggesting that religious practices or plant-based diets themselves promote unhealthy food consumption. Rather, household dietary practices may interact with the availability and affordability of culturally acceptable and nutritionally appropriate foods. Where such options are limited, practical food choices may become increasingly constrained [46,47]. This interpretation is consistent with the broader food–environment perspective emerging from the qualitative findings.

Access to maternal and child health services represented another important point of integration. Mothers who did not meet the recommended number of ANC visits had higher odds of both ZVF and UFC. Qualitative accounts of disrupted vaccination services, reduced access to health personnel, and fewer opportunities to obtain child-feeding information complemented these quantitative findings by illustrating the broader disruption of maternal–child health and nutrition services. In ethnic minority and conflict-affected areas of Myanmar, longstanding limitations in healthcare access have been further intensified by political instability and disruptions following the 2021 military coup [12,48,49]. ANC, immunization, and other maternal–child health visits can provide important opportunities for IYCF counseling and nutrition information. Maintaining access to these services may therefore have benefits that extend beyond clinical care by sustaining opportunities for child-feeding support and preventive nutrition guidance [50].

Taken together, the integrated findings suggest that unhealthy feeding practices in this IDP setting cannot be understood solely as individual maternal choices. Child developmental stage, household composition, economic constraints, time demands, household dietary practices, local food availability, and access to health and nutrition services all contributed to the context in which feeding decisions were made. The qualitative findings were particularly useful in contextualizing the quantitative associations involving child age, household structure, household income, and ANC access, while expanding understanding of paternal employment and the food environment. In contrast, the association with maternal decision-making autonomy remained unexplained qualitatively, underscoring the importance of distinguishing between statistically associated factors and mechanisms directly supported by the integrated data.

Some limitations should be considered when interpreting these findings. First, the cross-sectional design precludes causal inference. Additionally, ZVF and UFC were assessed based on food consumption during the previous day, as specified by the WHO/UNICEF IYCF indicators; therefore, these measures may not capture usual feeding patterns or day-to-day and seasonal variation. Recruitment through GMP sessions may have introduced selection bias by underrepresenting mothers with limited contact with health services, while the involvement of local nutrition volunteers in data collection may have increased the potential for socially desirable responses. Small subgroup sizes, particularly among low-income households and children aged 6–8 months, reduced the precision of some estimates. The regression models explained a limited proportion of the variance, and residual confounding by unmeasured structural and contextual factors, including conflict intensity, food availability, and humanitarian assistance, cannot be excluded. Participants were recruited from three geographical areas within a single IDP setting; although these areas were not treated as analytical clusters, some within-area dependence may remain. Eligibility was restricted to mothers who had lived with their husbands for at least two consecutive years because the study assessed maternal decision-making autonomy and husband supportive behavior within cohabiting households. This excluded single, widowed, separated, and other mothers not currently living with their husbands and may limit the generalizability of the findings to the broader maternal population in displacement settings. Qualitative participants were convenience-sampled from survey participants who voluntarily agreed to participate, introducing potential self-selection bias. Despite this limitation, the qualitative component was reported in accordance with the Consolidated Criteria for Reporting Qualitative Research (COREQ) to support transparent and complete reporting, with the completed checklist provided in Table A6. In addition, dichotomization of maternal decision-making autonomy and husband support using sample-dependent cutoffs may have resulted in information loss. The qualitative findings did not directly explain the associations involving maternal autonomy and paternal employment; therefore, the proposed mechanisms should not be interpreted as established pathways. Finally, sweetened beverage consumption was not assessed, limiting the characterization of unhealthy feeding practices.

## 5. Conclusions

These findings highlight the need for context-sensitive approaches to improve child feeding in displacement settings. Strengthening accessible community-based IYCF counseling and maintaining maternal and child health-service contact may help sustain opportunities for nutrition support when formal services are disrupted. Interventions should also consider the broader household and food–environment constraints affecting feeding practices, including food availability, affordability, caregiving demands, and household influences on feeding decisions. Supporting maternal decision-making should therefore be accompanied by access to appropriate nutrition information and feasible, nutritious food choices within the local displacement context.

## Figures and Tables

**Table 1:** Prevalence and characteristics of internally displaced mothers, children aged 6–23 months, and households, Kayin State, Myanmar, 2023 (*n* = 617).

Characteristics	Frequency	Percentage
A. Prevalence of Unhealthy Feeding Practices		
Zero Vegetable or Fruit Consumption		
Yes	180	29.2
No	437	70.8
Unhealthy Food Consumption		
Yes	355	57.5
No	262	42.5
B. General Characteristics of Mothers of Children Aged 6–23		
Maternal Age Group		
18–24 years	176	28.5
25–34 years	297	48.2
35 years and above	144	23.3
Mean ± SD ^1^	29 ± 5.9	
Min–Max	18–40	
Maternal Education		
Able to Read and Write	426	69.0
Unable	191	31.0
Maternal Employment Status		
Employed	208	33.7
Unemployed	409	66.3
Frequency of Antenatal Care Received		
Met with WHO recommendation ^2^	231	37.4
Not met	386	62.6
Postnatal IYCF ^3^ Counselling		
Received	363	58.8
Not received	254	41.2
Maternal Intrahousehold Decision-making Autonomy		
Higher	519	84.1
Lower	98	15.9
Husband’s Employment Status		
Employed	503	81.5
Unemployed	114	18.5
Husbands’ Supportive Behaviors for Child Feeding		
Higher	325	52.7
Lower	292	47.3
C. Characteristics of Children Aged 6–23 months		
Age Group		
6–8 months	40	6.5
9–11 months	57	9.2
12–23 months	520	84.3
Mean ± SD ^1^	18.5 ± 5.3	
Min–Max	6–23	
Sex		
Male	308	49.9
Female	309	50.1
Immunization Status		
Up to date	30	4.9
Not Up to date	587	95.1
History of Malnutrition		
Yes	33	5.3
No	584	94.7
D. Household Characteristics		
Household Type		
Nuclear	433	70.2
Extended	184	29.8
Monthly Income		
>250,000 Myanmar Kyats (MMK)	583	94.5
250,000 MMK and less	34	5.5
Household Dietary Practice		
Religion-influenced plant-based dietary practice	371	60.1
Conventional dietary practice	246	39.9

^1^ Standard deviation; ^2^ at least eight contacts; ^3^ infant and young child feeding.

**Table 2:** Determinants of ZVF and UFC practices among mothers of children aged 6–23 months in an IDP camp, Kayin State, Myanmar, 2023 (*n* = 617).

Variables	Zero Vegetable or Fruit Consumption (ZVF)				Unhealthy Food Consumption (UFC)			
	Binary Logistic Regression		Multivariable Logistic Regression ^†^		Binary Logistic Regression		Multivariable Logistic Regression ^††^	
	COR ^1^ (95% CI ^2^)	*p*-Value	AOR ^3^ (95% CI ^2^)	*p*-Value	COR ^1^ (95% CI ^2^)	*p*-Value	AOR ^3^ (95% CI ^2^)	*p*-Value
Frequency of Antenatal Care Received								
Met with WHO’s recommendation	Ref		Ref		Ref		Ref	
Not met	1.838 (1.260–2.682)	0.002 *	1.968 (1.297–2.988)	0.005 *	1.955 (1.391–2.746)	<0.001 *	1.830 (1.265–2.649)	0.001 *
Maternal Employment Status								
Unemployed	Ref		Ref		Ref		Ref	
Employed	1.047 (0.726–1.510)	0.805	1.309 (0.851–2.014)	0.221	1.494 (1.060–2.106)	0.022 *	1.144 (0.771–1.700)	0.504
Maternal Education								
Able to Read and Write	Ref		Ref		Ref		Ref	
Unable	1.066 (0.731–1.554)	0.742	1.496 (0.954–2.347)	0.079	1.163 (0.825–1.641)	0.389	1.144 (0.771–1.700)	0.504
Mother Decision-making Autonomy								
Lower	Ref		Ref		Ref		Ref	
Higher	1.611 (1.016–2.554)	0.043 *	1.757 (1.031–2.995)	0.038 *	1.412 (1.012–2.088)	0.013 *	1.606 (1.040–2.481)	0.033 *
Children Age Group								
12–23 months	Ref		Ref		3.270 (1.649–6.484)	<0.001 *	3.045 (1.468–6.318)	0.003 *
6–8 months	5.474 (2.912–11.342)	<0.001 *	7.570 (3.594–15.945)	<0.001 *	Ref		Ref	
9–11 months	2.785 (1.595–4.862)	<0.001 *	2.806 (1.530–5.146)	<0.001 *	1.510 (0.649–3.517)	0.339	1.512 (0.620–3.688)	0.364
Children Sex								
Male	Ref		Ref		Ref		Ref	
Female	1.165 (0.823–1.649)	0.390	1.298 (0.886–1.901)	0.180	0.954 (0.693–1.312)	0.771	0.889 (0.630–1.256)	0.505
Malnutrition History								
No	Ref		Ref		Ref		Ref	
Yes	2.116 (1.042–4.298)	0.038 *	2.086 (0.956–4.552)	0.065	0.680 (0.337–1.373)	0.282	0.705 (0.327–1.520)	0.372
Husband’s Employment Status								
Unemployed	Ref		Ref		Ref		Ref	
Employed	0.869 (0.560–1.349)	0.532	0.827 (0.500–1.368)	0.459	2.060 (1.365–3.110)	<0.001 *	1.788 (1.133–2.822)	0.013 *
Husband’s Supportive Behavior in child feeding								
Higher	Ref		Ref		Ref		Ref	
Lower	1.149 (0.772–1.709)	0.494	0.937 (0.603–1.456)	0.772	1.165 (0.811–1.673)	0.410	1.055 (0.707–1.573)	0.794
Household Dietary Practice								
Conventional dietary practices	Ref		Ref		Ref		Ref	
Religion-influenced plant-based dietary practice	1.984 (1.395–2.822)	<0.001 *	1.840 (1.249–2.710)	0.002 *	2.545 (1.827–3.546)	<0.001 *	2.566 (1.799–3.662)	<0.001 *
Type of Household								
Nuclear	Ref		Ref		Ref		Ref	
Extended	1.543 (1.033–2.306)	0.034 *	3.280 (1.006–6.698)	0.048 *	0.829 (0.284–2.425)	0.732	0.679 (0.203–2.272)	0.530
Household Monthly Income								
>250,000 MMK ^4^	Ref		Ref		2.292 (1.126–4.668)	0.022 *	1.993 (0.900–4.413)	0.089
250,000 MMK and less	3.320 (1.647–6.692)	<0.001 *	2.840 (1.291–6.248)	0.009 *	Ref		Ref	

^1^ Crude odds ratio; ^2^ 95% confidence interval; ^3^ adjusted odds ratio; ^4^ Myanmar kyats: * significant at *p*-value less than 0.05; ^†^ Nagelkerke R^2^—0.194, Hosmer–Lemeshow *p*-value—0.005, omnibus test *p*-value—<0.001; ^††^ Nagelkerke R^2^—0.162, Hosmer–Lemeshow *p*-value—0.131, omnibus test *p*-value—<0.001.

**Table 3:** Joint display integrating quantitative and qualitative findings.

Outcome	Quantitative Findings	Related Qualitative Findings	Integration Type ^2^	Meta-Inference
ZVF	Younger child age: 6–8 and 9–11 months had higher odds of ZVF than children aged 12–23 months	Theme 1: Familiarity Breeds Acceptance	Complementary	Mothers’ accounts of food rejection and unfamiliarity with tastes and textures provide context for the higher odds of ZVF among younger children during the early complementary-feeding period.
	Extended household structure was associated with higher odds of ZVF	Theme 2: Traditional Beliefs and Food Restrictions ^††^	Complementary	Accounts of family influence and food restrictions provide contextual insight into how household norms may shape feeding decisions in extended households, without establishing these practices as the mechanism underlying ZVF.
	Lower household income was associated with higher odds of ZVF	Theme 4: Constrained Local Food Environments ^†^	Complementary	Financial and geographical barriers to obtaining diverse nutritious foods provide context for the association between lower household income and ZVF.
UFC	Paternal employment was associated with higher odds of UFC	Theme 3: Time Constraints and Convenience Feeding	Expansion	Accounts of household time constraints and convenience feeding provide broader context for UFC but do not directly explain the association with paternal employment.
	Religion-influenced plant-based dietary practice was associated with higher odds of UFC compared with conventional dietary practice	Theme 4: Constrained Local Food Environments ^†^	Expansion	Qualitative findings broaden understanding of the constrained food environment in which household dietary practices occur but do not directly establish why religion-influenced plant-based dietary practice was associated with UFC.
	Children aged 12–23 months had higher odds of UFC than children aged 6–8 months	Theme 5: Preference for Sweet Foods	Complementary	Mothers’ accounts of increasing food preferences and requests for sweet foods as children grew older provide context for the higher odds of UFC among older children.
ZVF and UFC	Mothers who did not meet the WHO-recommended number of ANC ^1^ contacts had higher odds of both ZVF and UFC	Theme 6: Disrupted Access to Health and Nutrition Services *	Complementary	Reports of disrupted access to maternal-child health and nutrition services provide context for the association between inadequate ANC contacts and unhealthy feeding practices by illustrating reduced opportunities for nutrition information and counselling.
	Higher maternal intrahousehold decision-making autonomy was associated with higher odds of ZVF and UFC	No corresponding qualitative theme emerged	Silence	The quantitative association between higher maternal autonomy and ZVF/UFC was not directly represented in the qualitative findings; therefore, the qualitative phase did not provide an explanation for this association.

^1^ Antenatal care; ^2^ adapted from established mixed-method integration frameworks; complementarity-qualitative findings elaborate or enrich quantitative findings; expansion—qualitative findings extend beyond the quantitative findings; silence—no corresponding qualitative theme emerged to support quantitative findings; * structural determinants; ^†^ food environmental determinants; ^††^ sociocultural determinants.

## Data Availability

The data generated and/or analyzed during this study are included in this published article. Other data supporting the findings of this study are available from the corresponding author upon request.

## Abbreviations

The following abbreviations are used in this manuscript:

CIWA	Composite Index of Women’s Autonomy
COREQ	Consolidated Criteria for Reporting Qualitative Research
GMP	Growth Monitoring and Promotion
IDP	Internally Displaced Person
IYCF	Infant and Young Child Feeding
NHDF	Nutrition Health Disparities Framework
UFC	Unhealthy Food Consumption
ZVF	Zero Vegetable or Fruit Consumption

## Appendix A. Operational Definitions and Calculation Formulas

### Appendix A.1. Zero Vegetable or Fruit Consumption; ZVF

It is defined as the proportion of children aged 6–23 months who did not consume any vegetables or fruits during the previous day.

Calculation of ZVF Prevalence

$$ZVF=\frac{\begin{array}{c} Number\;of\;children\;aged\;6–23\;months\;who\;did\;not\;consume\;\\ any\;vegetables\;of\;fruits\;during\;the\;previous\;day \end{array}}{Number\;of\;children\;aged\;6–23\;months}$$

### Appendix A.2. Unhealthy Food Consumption: UFC

It is defined as the proportion of children aged 6–23 months who consumed selected sentinel unhealthy foods during the previous day.

Calculation of UFC Prevalence

$$UFC=\frac{\begin{array}{c} Number\;of\;children\;aged\;6–23\;months\;consumed\;selected\;\\ sentinel\;unhealthy\;foods\;during\;the\;previous\;day \end{array}}{Number\;of\;children\;aged\;6–23\;months}$$

### Appendix A.3. Selected Sentinel Unhealthy Foods

Those foods are defined as the food items likely consumed by infants and young children and prepared high in added sugar, salt, and/or unhealthy fats. They include: (1) candies, chocolates, and sugar confections, (2) frozen treats, (3) sweet baked or fried confections, and (4) fried, salty, or refined-carbohydrate snacks.

## Appendix B

**Table A1:** Sensitivity analyses using mean- and median-based cut-offs, and continuous score for maternal decision-making autonomy and husband support (*n* = 617).

Outcome	Predictor	Mean Cutoff AOR (95% CI)	*p*-Value	Median Cutoff AOR (95% CI)	*p*-Value	Continuous Score AOR (95% CI)	*p*-Value	Consistency
ZVF	Higher Maternal Autonomy	1.757 (1.031–2.995)	0.038	1.737 (1.013–2.978)	0.045	1.109 (1.002–1.227)	0.046	Consistent
UFC	Higher Maternal Autonomy	1.606 (1.040–2.481)	0.033	1.517 (1.211–2.353)	0.042	1.096 (1.011–1.187)	0.026	Consistent
ZVF	Lower Husband Support	0.937 (0.603–1.456)	0.772	1.574 (0.882–2.810)	0.124	1.043 (0.939–1.157)	0.434	Consistent (no association)
UFC	Lower Husband Support	1.055 (0.707–1.573)	0.794	1.187 (0.791–1.789)	0.521	1.116 (0.982–1.269)	0.093	Consistent (no association)

Note: Consistency was assessed based on whether the direction, magnitude, and overall inference (statistically significant or non-significant) remained unchanged between the primary (mean cut-off) and sensitivity (median cut-off and continuous score) analyses.

**Table A2:** Comparison of alternative multivariable logistic regression models for ZVF (*n* = 617).

Variables	Multivariable Logistic Regression	
	Statistically Selected Model ^a^	Expanded Theory-Informed Model ^b^
	AOR (95% CI)	AOR (95% CI)
Frequency of Antenatal Care Received		
Met with WHO’s recommendation	Ref	Ref
Not met	1.784 (1.192–2.668)	1.968 (1.297–2.988)
Maternal Employment Status		
Unemployed		Ref
Employed		1.309 (0.851–2.014)
Maternal Education		
Able to Read and Write		Ref
Unable		1.496 (0.954–2.347)
Mother Decision-making Autonomy		
Lower	Ref	Ref
Higher	1.891 (1.121–3.192)	1.757 (1.031–2.995)
Children Age Group		
12–23 months	Ref	Ref
6–8 months	6.545 (3.177–13.485)	7.570 (3.594–15.945)
9–11 months	2.471 (1.372–4.452)	2.806 (1.530–5.146)
Children Sex		
Male		Ref
Female		1.298 (0.886–1.901)
Malnutrition History		
No	Ref	Ref
Yes	1.894 (0.880–4.077)	2.086 (0.956–4.552)
Husband’s Employment Status		
Unemployed		Ref
Employed		0.827 (0.500–1.368)
Husband’s Supportive Behavior in child feeding		
Higher		Ref
Lower		0.937 (0.603–1.456)
Household Dietary Practice		
Conventional dietary practices	Ref	Ref
Religion-influenced plant-based dietary practice	1.804 (1.234–2.638)	1.840 (1.249–2.710)
Type of Household		
Nuclear	Ref	Ref
Extended	1.734 (1.127–2.669)	3.280 (1.006–6.698)
Household Monthly Income		
>250,000 MMK ^1^	Ref	Ref
250,000 MMK and less	2.849 (1.319–6.153)	2.840 (1.291–6.248)

^a^ Statistically Selected Model: included variables meeting the prespecified *p* < 0.20 criterion in univariable analysis and was examined as an alternative specification in sensitivity analysis; Nagelkerke R^2^ = 0.177, Hosmer–Lemeshow *p* = 0.103, Omnibus test *p* < 0.001; ^b^ Expanded theory-informed Model: additionally included maternal employment, maternal education, child sex, husband’s employment status, and husband’s supportive behaviour in child feeding based on their conceptual relevance and plausible confounding relationships; Nagelkerke R^2^ = 0.194, Hosmer–Lemeshow *p* = 0.005, Omnibus test *p* < 0.001; ^1^ Myanmar Kyats.

**Table A3:** Coding tree showing the development of initial codes, minor themes, and major themes from the deductive thematic analysis.

Initial Codes	Minor Theme	Derivation of Theme	Major Theme
Refused new foods; preferred breastfeeding; rejected unfamiliar taste or texture; gradually accepted complementary foods	Acceptance of familiar foods	Derived inductively from repeated accounts of children’s reluctance to accept unfamiliar complementary foods and increasing acceptance through repeated exposure.	Theme 1. Familiarity Breeds Acceptance
Avoided vegetables because of food taboos; avoided meat due to traditional beliefs; grandparent influence; religious dietary restrictions	Traditional food restrictions	Developed from recurrent discussions of family traditions, cultural beliefs, and religious dietary practices shaping child feeding decisions.	Theme 2. Traditional Beliefs and Food Restrictions
Busy with household chores; caring for multiple children; limited time for food preparation; part-time work; reliance on ready-made foods	Maternal workload and convenience feeding	Derived from repeated descriptions of competing household responsibilities leading mothers to prioritise convenient feeding practices.	Theme 3. Time Constraints and Convenience Feeding
Distant markets; irregular mobile vendors; limited food availability; dependence on food rations; foraging; home gardening	Physical and economic food constraints	Developed from participants’ descriptions of limited food availability, affordability, and accessibility, together with adaptive food acquisition strategies.	Theme 4. Constrained Local Food Environment
Child preferred sweet snacks; attractive packaging; readily available processed foods; biscuits and cakes as convenient foods	Preference for unhealthy processed foods	Derived from recurrent accounts of children’s preference for sweet, processed foods and caregivers’ reliance on convenient packaged snacks.	Theme 5. Preference for Sweet Foods
Reduced access to health services after conflict; interrupted vaccination; fewer health education opportunities; language barriers; reliance on healthcare workers	Limited access to health and nutrition services	Developed from participants’ experiences of disrupted health and nutrition services, reduced opportunities for nutrition counselling, and communication barriers following the conflict.	Theme 6. Disrupted Access to Health and Nutrition Services

Note: Themes were developed using a deductive thematic analysis informed by the quantitative findings and the semi-structured interview guide. Initial codes were grouped into conceptually related categories (minor themes), which were iteratively reviewed and refined into the final major themes through regular discussions among the research team until consensus was reached.

**Table A4:** ZVF and UFC among the study population, Kayin State, Myanmar, 2023 (*n* = 617).

Variables	Total *n* (%)	ZVF		UFC	
		Yes *n* (%)	No *n* (%)	Yes *n* (%)	No *n* (%)
Frequency of Antenatal Care Received					
Met with WHO’s recommendation	231 (37.4)	50 (21.6)	181 (78.4)	110 (47.6)	121 (52.4)
Not met	386 (62.6)	130 (33.7)	256 (66.3)	245 (63.5)	141 (36.5)
Maternal Employment Status					
Unemployed	409 (66.3)	118 (28.9)	291 (71.1)	222 (54.3)	187 (45.7)
Employed	208 (33.7)	62 (29.8)	146 (70.2)	133 (63.9)	75 (36.1)
Maternal Education					
Able to Read and Write	426 (69.0)	126 (29.6)	300 (70.4)	250 (58.7)	176 (41.3)
Unable	191 (31.0)	54 (28.3)	137 (71.7)	105 (55.0)	86 (45.0)
Mother Decision-making Autonomy					
Lower	98 (15.9)	20 (20.4)	78 (79.6)	46 (46.9)	52 (53.1)
Higher	519 (84.1)	160 (30.8)	359 (69.2)	309 (59.5)	210 (40.5)
Children Age Group					
6–8 months	40 (6.5)	26 (65.0)	14 (35.0)	13 (32.5)	27 (67.5)
9–11 months	57 (9.2)	27 (47.4)	30 (52.6)	24 (42.1)	33 (57.9)
12–23 months	520 (84.3)	127 (24.4)	393 (75.6)	318 (61.2)	202 (38.8)
Children Sex					
Male	308 (49.9)	85 (27.6)	223 (72.4)	179 (58.1)	129 (41.9)
Female	309 (50.1)	95 (30.7)	214 (69.3)	176 (57.0)	133 (43.0)
Malnutrition History					
No	584 (94.7)	165 (28.3)	419 (71.7)	339 (58.0)	245 (42.0)
Yes	33 (5.3)	15 (45.5)	18 (54.5)	16 (48.5)	17 (51.5)
Husband’s Employment Status					
Unemployed	114 (18.5)	36 (31.6)	78 (68.4)	49 (43.0)	65 (57.0)
Employed	503 (81.5)	144 (28.6)	359 (71.4)	306 (60.8)	197 (39.2)
Husband’s Supportive Behavior in child feeding					
Higher	325 (52.6)	91 (28.0)	234 (72.0)	198 (60.9)	127 (39.1)
Lower	292 (47.4)	89 (30.5)	203 (69.5)	157 (53.8)	135 (46.2)
Household Dietary Practice					
Conventional dietary practices	246 (39.9)	52 (21.1)	194 (78.9)	108 (43.9)	138 (56.1)
Religion-influenced plant-based dietary practice	371 (60.1)	128 (34.5)	243 (65.5)	247 (66.6)	124 (33.4)
Type of Household					
Nuclear	433 (70.2)	110 (25.4)	323 (74.6)	253 (58.4)	180 (41.6)
Extended	184 (29.8)	70 (38.0)	114 (62.0)	102 (55.4)	82 (44.6)
Household Monthly Income					
>250,000 MMK ^1^	583 (94.5)	161 (27.6)	422 (72.4)	342 (58.7)	241 (41.3)
250,000 MMK and less	34 (5.5)	19 (55.9)	15 (44.1)	13 (38.2)	21 (61.8)

^1^ Myanmar Kyats.

**Table A5:** Participants’ characteristics of focus group discussion (*n* = 50).

Characteristics	Frequency	Percentage
Mothers’ Age Group		
20 years and younger	3	6.0
21–30 years	32	64.0
31 years and older	15	30.0
Median (Q1–Q3)	25 (22.5–32.5)	
Min Max	19–35	
Education—Karen Ethnic Language		
Able to Read and Write	14	28.0
Not	36	72.0
Education—Barma Language		
Able to Read and Write	8	16.0
Not	42	84.0
Employment		
Employed	11	22.0
Unemployed	39	78.0
Children Age Group		
6–8 months	7	14.0
9–11 months	11	22.0
12–23 months	32	64.0
Mean ± SD ^1^	14.7 ± 5.06	
Min-Max	6–23	

^1^ Standard Deviation.

**Table A6:** Consolidated criteria for reporting qualitative studies (COREQ): 32-item checklist.

No. Item	Guide Questions/Description	Reported on Page Number
Domain 1: Research team and reflexivity		
Personal Characteristics		
1. Interview/Facilitator	Which author/s conducted the interview or focus group?	7–8
2. Credentials	What were the researcher’s credentials? e.g., PhD, MD	7–8
3. Occupation	What was their occupation at the time of the study?	7–8
4. Gender	Was the researcher male or female?	7–8
5. Experience and Training	What experience or training did the researcher have?	7–8
Relationship with Participants		7–8
6. Relationship established	Was a relationship established prior to study commencement?	7–8
7. Participant knowledge of the interview	What did the participants know about the researcher? e.g., personal goals, reasons for doing the research	7–8
8. Interviewer Characteristics	What characteristics were reported about the inter viewer/facilitator? e.g., Bias, assumptions, reasons and interests in the research topic	7–8
Domain 2: Study Design		
Theoretical Framework		
9. Methodological orientation and Theory	What methodological orientation was stated to underpin the study? e.g., grounded theory, discourse analysis, ethnography, phenomenology, content analysis	
Participant Selection		
10. Sampling	How were participants selected? e.g., purposive, convenience, consecutive, snowball	7–8
11. Method of Approach	How were participants approached? e.g., face-to-face, telephone, mail, email	7–8
12. Sample Size	How many participants were in the study?	7–8
13. Non-participation	How many people refused to participate or dropped out? Reasons?	NA
Setting		
14. Setting of data collection	Where was the data collected? e.g., home, clinic, workplace	7–8
15. Presence of non-participants	Was anyone else present besides the participants and researchers?	7–8
16. Description of sample	What are the important characteristics of the sample? e.g., demographic data, date	Table A4
Data Collection		
17. Interview Guide	Were questions, prompts, guides provided by the authors? Was it pilot tested?	7–8
18. Repeat Interview	Were repeat inter views carried out? If yes, how many?	NA
19. Audio/Visual Recording	Did the research use audio or visual recording to collect the data?	7–8
20. Field Notes	Were field notes made during and/or after the interview or focus group?	7–8
21. Duration	What was the duration of the inter views or focus group?	7–8
22. Data Saturation	Was data saturation discussed?	7–8
23. Transcripts returned	Were transcripts returned to participants for comment and/or correction?	7–8
Domain 3: Analysis and Findings		
24. Number of Data Coders	How many data coders coded the data?	7–8
25. Description of the coding tree	Did authors provide a description of the coding tree?	Table A3
26. Derivation of themes	Were themes identified in advance or derived from the data?	Table A3
27. Software	What software, if applicable, was used to manage the data?	7
28. Participant Checking	Did participants provide feedback on the findings?	7
Reporting		
29. Quotations presented	Were participant quotations presented to illustrate the themes/findings? Was each quotation identified? e.g., participant number	14–16
30. Data and Findings Consistent	Was there consistency between the data presented and the findings?	14–16
31. Clarity of Major Themes	Were major themes clearly presented in the findings?	14–16
32. Clarity of Minor Themes	Is there a description of diverse cases or discussion of minor themes?	Table A3

## Appendix C. Semi-Structured Focus Group Discussion Guide

Introduction

Thank you for participating in this focus group discussion. We are interested in learning about your experiences and opinions regarding infant and young child feeding (IYCF) practices for children aged 6–23 months living in this camp. There are no right or wrong answers. We encourage everyone to share their experiences freely. With your permission, this discussion will be audio-recorded, and all information will remain confidential and used only for research purposes.

Theme 1. Current Infant and Young Child Feeding Practices

1. Please describe how you usually feed your child during a typical day.
2. What types of foods does your child usually eat? Why do you choose these foods?
3. Can you describe your experience introducing complementary foods?
4. What helps or makes it difficult to provide vegetables, fruits, eggs, or meat to your child?
5. What influences how often you feed your child each day?
6. What kinds of snacks or processed foods does your child usually eat? Why?

Probes: Food availability, Child food preference, Family food practices, Seasonal variation, and Cost of foods.

Theme 2. Factors Influencing Child Feeding Practices

Maternal decision-making

1. How do decisions within your household affect what your child eats?
2. Can you describe situations where you decide independently or together with your husband about food or healthcare?

Husband and family support

1. How does your husband support (or not support) feeding your child?
2. Are there other family members who influence your child’s feeding? How?

Household and community environment

1. What challenges do you face in obtaining healthy foods for your child?
2. How do income, work responsibilities, or household size affect child feeding?

Probes: Household responsibilities, Food affordability, Market accessibility, Religious or cultural dietary practices, and Community food environment

Theme 3. Health Services and Child Feeding

1. How have antenatal care, postnatal care, or child health services influenced your feeding practices?
2. What nutrition information have you received from healthcare workers?
3. What makes it easy or difficult to follow this advice?

Probes: Health education, Vaccination visits, Language barriers, and Access to services after displacement

Closing Question: Is there anything else you would like to share about feeding young children in this community that we have not discussed?
